# Beyond factor analysis: Multidimensionality and the Parkinson’s Disease Sleep Scale-Revised

**DOI:** 10.1371/journal.pone.0192394

**Published:** 2018-02-12

**Authors:** Maria E. Pushpanathan, Andrea M. Loftus, Natalie Gasson, Meghan G. Thomas, Caitlin F. Timms, Michelle Olaithe, Romola S. Bucks

**Affiliations:** 1 School of Psychological Sciences, The University of Western Australia, Crawley, Western Australia, Australia; 2 School of Psychology and Speech Pathology, Curtin University, Bentley, Western Australia, Australia; 3 School of Medical Sciences, Edith Cowan University, Joondalup, Western Australia, Australia; Radboud Universiteit, NETHERLANDS

## Abstract

Many studies have sought to describe the relationship between sleep disturbance and cognition in Parkinson’s disease (PD). The Parkinson’s Disease Sleep Scale (PDSS) and its variants (the Parkinson’s disease Sleep Scale-Revised; PDSS-R, and the Parkinson’s Disease Sleep Scale-2; PDSS-2) quantify a range of symptoms impacting sleep in only 15 items. However, data from these scales may be problematic as included items have considerable conceptual breadth, and there may be overlap in the constructs assessed. Multidimensional measurement models, accounting for the tendency for items to measure multiple constructs, may be useful more accurately to model variance than traditional confirmatory factor analysis. In the present study, we tested the hypothesis that a multidimensional model (a bifactor model) is more appropriate than traditional factor analysis for data generated by these types of scales, using data collected using the PDSS-R as an exemplar. 166 participants diagnosed with idiopathic PD participated in this study. Using PDSS-R data, we compared three models: a unidimensional model; a 3-factor model consisting of sub-factors measuring insomnia, motor symptoms and obstructive sleep apnoea (OSA) and REM sleep behaviour disorder (RBD) symptoms; and, a confirmatory bifactor model with both a general factor and the same three sub-factors. Only the confirmatory bifactor model achieved satisfactory model fit, suggesting that PDSS-R data are multidimensional. There were differential associations between factor scores and patient characteristics, suggesting that some PDSS-R items, but not others, are influenced by mood and personality in addition to sleep symptoms. Multidimensional measurement models may also be a helpful tool in the PDSS and the PDSS-2 scales and may improve the sensitivity of these instruments.

## Introduction

A number of factors act to disrupt sleep in Parkinson’s disease (PD), including primary sleep disorder, such as insomnia or REM sleep behaviour disorder (RBD) and sleep disturbance secondary to the symptoms of PD (e.g. dystonia, rigidity or medication effects). The Parkinson’s Disease Sleep Scale (PDSS) [1] and its variants (the Parkinson’s disease Sleep Scale-Revised; PDSS-R, [2] and the Parkinson’s Disease Sleep Scale-2; PDSS-2 [3]) measure a range of symptoms that commonly disrupt sleep in PD. These scales have, therefore, been widely implemented. Research applications have been varied including, for example, the exploration of how non-motor symptoms interact, [4] and outcome measures in clinical trials. [5,6]

Data derived from the PDSS scales may, however, be problematic as they measure a large number of symptoms in relatively few items. [7] The published factor analyses of the PDSS and PDSS-2 have all have employed Principal Components Analysis, [3,8,9] precluding evaluation of model fit. Moreover, evidence suggests that the development and experience of PD symptoms vary in different types of patients. For example, motor and non-motor symptoms diverge in men and women. Objective symptom assessment has shown men tend to be more severely affected while women are more open to reporting difficulty. [10]

Taken together, these issues are of concern. As the PDSS scales are now ubiquitous, used widely both in clinical practice and medication trials, it is critical that we understand the degree to which these scales measure sleep and the ways in which they are vulnerable to systematic bias. To examine these issues, we first need a robust factor model.

Considering both the nature of the PDSS scales and the population for which they were designed, a multidimensional measurement model may be appropriate for the analysis of PDSS data. While traditional factor analytic techniques assume that each item measures a single construct, multidimensional measurement models allow items simultaneously to measure more than one construct. Morin, Arens and Marsh describe the concept of construct relevant multidimensionality, ‘…items are very seldom pure indicators of the constructs they are purported to measure. Rather, they tend to be fallible indicators including at least some degree of relevant association with constructs other than the main constructs that they are designed to measure.’. ([11]; p.6) A bifactor model partials out covariance common to all scale items into a single, ‘general’ factor while simultaneously identifying sub-factors, sharing unique covariances, as one would using traditional factor analysis. [11]

Variables such as disease duration, severity, medication, sex, age, personality and mood may be important sources of construct relevant multidimensionality in PDSS items. These scales, however, have not yet been examined within a multidimensional measurement framework. The examination of associations between PDSS factors and the aforementioned parameters will inform about the interplay between patient characteristics and different aspects of sleep in PD.

The present study tests the hypothesis that data from the PDSS scales are best modelled using a multidimensional technique using data collected via the Parkinson’s Sleep Scale-Revised. [2] Models for comparison were determined by examining how PDSS scales are currently used. Total scale scores are most frequently reported, ergo the first model tested was a single factor confirmatory factor analysis (CFA). We then identified the theoretical constructs assessed by the PDSS-R: 1. Insomnia; 2. Motor symptoms disturbing sleep; and, 3. obstructive sleep apnoea (OSA) and REM sleep behaviour disorder (RBD) symptoms. Items measuring these two disorders were clustered together as they frequently co-occur in PD [12] and symptoms may overlap (severe OSA may present as pseudo-RBD). [13] Thus, the present study compares the fit of three models: a 1 factor CFA; a 3 factor CFA; and, an orthogonal confirmatory bifactor model with the one general and three specific factors combined. Associations between factor scores from the best fitting model and sample parameters (age, sex, disease duration, severity, medication, neuroticism, and mood) were then examined.

## Methods

The study was approved by the Human Research Ethics Committees of the University of Western Australia, Edith Cowan University, and Curtin University. All participants provided written, informed consent.

### Participants

PDSS-R data were collected as part of a longitudinal study examining disease progression in a community cohort of people with PD. [14] One hundred and eighty-six participants with idiopathic PD (diagnosed by a neurologist or geriatrician in accordance with United Kingdom Parkinson’s Disease Society Brain Bank Clinical Criteria) [15] completed the scale. Participants were recruited through Parkinson’s Western Australia community events, support groups, advertisements and referral from health professionals. Participants with significant cognitive impairment (< 24 on the Mini-Mental State Examination; *N* = 11) or neurological co-morbidities (*N* = 9) were excluded, leaving 166.

#### Measures

Demographic data were collected, including medical history, medication, age, and date of diagnosis. Neuroticism was measured using the Big Five Aspects Scale (BFAS). [16] Mood was measured using the total score of the short form Depression, Anxiety and Stress Scale (DASS-21). [17]

**The Parkinson’s Disease Sleep Scale-Revised (PDSS-R)**. The PDSS-R is a 15-item, self-administered scale in which patients rate aspects of sleep affected in PD. Participants respond by placing a mark on a visual analogue line, where 0 indicates ‘excellent’ or ‘never’ and 10 indicates ‘awful’ or ‘always.’ The PDSS-R measures overall sleep quality, sleep initiation and maintenance, nocturia, sleep disruption due to ‘wearing-off’ symptoms, restless limbs, RBD, OSA, dystonia, sleep refreshment, and sleep attacks. Higher scores indicate poorer sleep.

#### Analyses

Data screening, descriptive and correlational analyses were performed using SPSS, IBM (Version 22).

Missing data (<5%) were missing completely at random; Little’s MCAR χ^2^(56) = 55.34, *p* = .05. As the PDSS-R data were positively skewed and imputation methods assume a normal distribution, we did not impute missing values.

Models were created in Mplus for Windows (v7.2). The data did not meet the assumption of normality and our covariates were ordinal or skewed; therefore, we selected maximum likelihood with robust standard errors (MLR) as our model estimator in accordance with recommendations for modelling data of this type. Models were adjusted in accordance with modification indices, provided i) the model had close fit before adjustment; ii) the modification indices were >10, and iii) the suggested modifications were theoretically justifiable. [18]

Factor scores were correlated with age, disease duration, disease severity (Hoehn and Yahr score; H&Y), Levodopa equivalent dose (LED), mood, and neuroticism (BFAS neuroticism sub-scale score). Relationships between sex and factor scores were assessed using Mann-Whitney U tests.

## Results

Participants were 41–85 years of age (*M ± SD* 66.13 ± 9.29) with > 1–27 years’ disease duration (*M ± SD* 5.44 ± 4.97 years). Hoehn and Yahr scores ranged from 1–4 (*M ± SD* 1.83 ± 0.64), and 110 participants (66.3%) were male. Table 1 contains descriptive statistics for PDSS-R item scores.

**Table 1:** Means, standard deviations and standardised factor loadings for PDSS-R data.

Item	Mean (SD)	% of sample who did not endorse this item Score ≤ 1	General	Insomnia	OSA/ RBD symptoms	Motor Symptoms
1. Overall sleep quality	4.60 (2.66)	7.8%	0.49‡	0.72‡		
2. Difficulty falling asleep	2.96 (2.66)	32.5%	0.47‡	0.25†		
3. Difficulty staying asleep	4.91 (2.94)	10.9%	0.49‡	0.64‡		
4. Restless Limbs	3.58 (3.09)	29.3%	0.41‡			0.66
5. Violent Behaviour	1.80 (2.63)	66.3%	0.32‡		0.51‡	
6. Distressing dreams	1.99 (2.47)	55.4%	0.46‡		0.26†	
7. Snore loudly and breathing pauses (Both)	3.06 (3.21)	42.9%	0.23†		0.41‡	
8. Wake up gasping for air	.85 (1.61)	81.9%	0.52‡		0.68‡	
9. Difficulty going back to sleep because of stiffness, tremor, or slowness	2.93 (2.88)	41.0%	0.72‡			0.34
10. Nocturia	2.23 (2.91)	54.8%	0.55‡			
11. Painful muscle cramps	2.86 (2.86)	38.6%	0.57‡			0.13
12. Early morning painful posturing of arms or legs	2.61 (2.93)	48.2%	0.57‡			0.24
13. Waking tremor	2.44 (3.02)	54.8%	0.43‡			0.10
14. Unrefreshing sleep	3.28 (2.82)	29.5%	0.69‡	0.07		
15. Sleep attacks	2.56 (2.58)	40.2%	0.35‡			
Total Score	42.64 (23.08)	N/A	N/A	N/A	N/A	N/A

^†^ significant at .05 level;

^‡^ significant at .01 level

Model fit statistics are reported in Table 2. Fit indices for both the 1 and 3-factor CFAs suggest model misspecification while the confirmatory bifactor model had acceptable fit.

**Table 2:** Model fit indices for the 1 factor CFA, the 3 factor CFA and the confirmatory bifactor model.

	S-B χ^2^	S-B χ^2^/df	Δχ^2^(df)	RMSEA (90% CI)	CFI	SRMR
1 Factor CFA	320.24‡	3.56		0.13 (0.11–0.14)	0.63	0.09
3 Factor CFA	255.22‡	2.87	47.78(3)‡^a^	0.11 (0.09–0.12)	0.73	0.12
Confirmatory Bifactor Model	122.75‡	1.66	87.83(15)‡^b^	0.07 (0.04–0.08)	0.92	0.06

*Note*: S-B χ^2 =^ Satora-Bentler Chi Square; RMSEA = Root Mean Squared Error of Approximation; CFI = Comparative Fit Index; SRMR = standardised root mean square residual. Ideal values for fit indices; S-B χ^2^/df <3; RMSEA ≤.05; RMSEA 90% CI must cross 0.05; CFI ≥.95; SMSR ≤.08;

^‡^significant at *p <* .01;

^a^ significant compared to 1 factor CFA;

^b^ significant compared to 3 factor CFA

Three adjustments were made to the confirmatory bifactor model on the basis of modification indices. Error variances were allowed to covary between 3 item pairs: 5 (violent behaviours) and 6 (distressing dreams); 11 (muscle cramps) and 12 (painful posturing); and 9 (difficulty going back to sleep) and 15 (unexpectedly falling asleep), which covary negatively.

**Fig 1:**
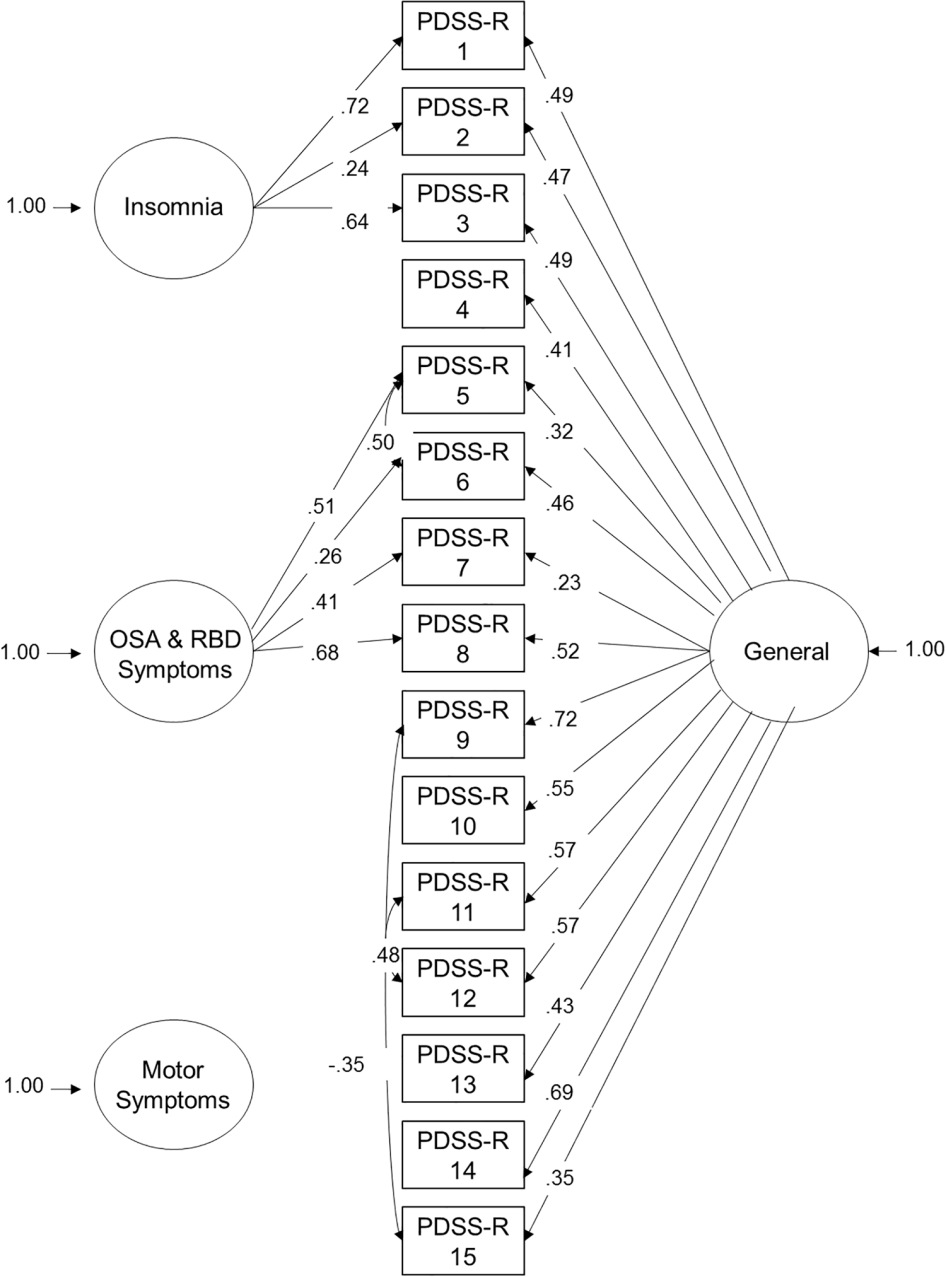
Diagram of confirmatory bifactor model: Regression coefficients for all significant paths. Squares represent measured variables, circles represent latent variables, curved lines are where we have allowed covariance of error terms to improve model fit.

The confirmatory bifactor model consists of a general factor, in addition to 3 sub-factors: insomnia, OSA and RBD symptoms and motor symptoms (see Fig 1). The non-significant factor loadings for the motor symptoms sub-factor (see Table 1) suggest that this factor does not exist outside of the general factor. Therefore, this sub-factor was not further analysed.

Individuals with higher general PDSS-R factor scores were more neurotic, reported poorer mood, longer disease duration, and higher LED than those with lower general factor scores (see Table 3 for Spearman’s Rho correlations). There were no sex differences (*U* = 2337.50, *p* = .18).

**Table 3:** Correlations between confirmatory bifactor model factor scores and participant characteristics.

	Insomnia	OSA/ RBD	Age	Duration	BFAS-Neuroticism	DASS-total	H&Y	LED
General	.26‡^a^^,^^b^	-.26‡^a^	-.02	.16†^c^	.34‡	.50‡^c^^,^^d^	-.06	.30‡^d^
Insomnia	1	-.22‡^b^	-.20†^e^	.12	.10	.17†^e^	-.03	.21†
OSA/ RBD	-.22‡^b^	1	.13	-.11	-.12	-.06	.16	-.07

Note: OSA = Obstructive sleep apnoea; BFAS = Big Five Aspect Scale; DASS = Depression Anxiety and Stress Scale; H&Y = Hoehn & Yahr Scale; LED = Levodopa Equivalent Dose;

^†^ significant at .05 level;

^‡^ significant at .01 level

^a,b,c^ Pair significantly different at .01 level (2 tailed)

^d,e^ Pair significantly different at .05 level (2 tailed)

Participants who reported more severe insomnia also reported poorer mood, were taking higher LED doses and were younger than those with lower insomnia scores. There were no sex differences (*U* = 2292.00, *p* = .13).

There were no associations with OSA and RBD symptom factor scores; although men had higher scores than women (*U* = 2375, *p* = .02).

Correlations were compared using software designed to test whether the magnitude of correlations derived from a single sample and which also share a dependent variable are significantly different. [19] Results are included in Table 3 and discussed below.

## Discussion

The PDSS was developed to measure common sleep disturbances in 2002 and has since been revised twice. The PDSS-R was developed in 2005 and proposed item changes including measurement of OSA and RBD. [2] The final revision of the scale, the PDSS-2, was published in 2011 and, while the scale retained the 15-item structure of the first two iterations, the PDSS-2 introduced a new response format (visual analogue to Likert), changed the direction of scoring (higher scores indicate poorer sleep) and amended some item content to include measurement of akinesia, pain, and restless leg syndrome. [3] The PDSS and the PDSS-2 have been widely taken up and used to quantify sleep disturbances across different populations and, importantly have been used as the outcome measure in clinical trials of interventions designed to improve sleep in PD. [6,20,21] Both the PDSS and the PDSS-2 were factor analysed using principal components analysis (PCA) and either the total score (i.e. assuming a one-factor structure) or a 3-factor structure (with three sub-scales) has been reported. [3,9,22] Despite its popularity, a significant limitation of PCA is the absence of model-fit statistics, precluding the comparison of alternative models. Where the PDSS measures have been used to quantify the relationship between sleep and other non-motor symptoms, results have been mixed. [22–24] We posit that despite evidence-based and comprehensive item content, the scales measure many sleep symptoms in relatively few items, and this feature may yield multidimensional data that may be more effectively be factor analysed using a model that accounts for multidimensionality. We, therefore, examined construct-relevant multidimensionality using a bifactor analysis of PDSS-R data as an exemplar.

This study is the first to apply a multidimensional model to any version of the Parkinson′s Disease Sleep Scale. One and three-factor traditional (confirmatory factor analysis) models were compared against a confirmatory bifactor model. The confirmatory bifactor model, consisting of a general factor (analogous to the one-factor or total score model) and three sub-factors (analogous to the three-factor model), best fit the PDSS-R data. Sleep-related motor symptoms did not exist as a sub-factor outside of the general factor.

When modelling data using bifactor accounts, a general factor draws on covariance from all items and is therefore thought to represent an overarching factor (i.e. sleep quality). However, the results of the correlational analyses are not wholly consistent with this view. If the general factor were measuring general sleep problems associated with PD, we might expect positive correlations with disease duration, disease severity, and LED: all of which are associated with deterioration of sleep quality in PD. [24] While the general factor was related to disease duration and LED, it was also associated more strongly with neuroticism, and *most* strongly with mood. There are two possible explanations for these observations. First, mood instability may be a confound, because of response bias- the general factor may reflect this. Typically, neuroticism and affective symptoms have been associated with disproportionally severe subjective sleep problems than would be expected based on objective assessment. [25] Moreover, as sleep symptoms feature prominently in the presentation of affective disorders; depression is likely to be comorbid with sleep disturbance (indeed, depression and disturbed sleep often occur in tandem as part of the non-motor symptom complex of PD). [26] In addition, premorbid neuroticism itself predicts PD [27] and is associated with poorer sleep in the general population. [28] Martinez- Martin et al. noted that depression and anxiety accounted for one-quarter of the variance in PDSS total scores. [29] General factor scores may be influenced by affective symptoms, and the concurrent administration of depression/anxiety scales may aid interpretation.

As anticipated, higher insomnia scores were associated with poorer mood and higher LED. The relationship between severe insomnia and younger age seemed counter-intuitive in light of the broader sleep literature. (see reviews: [30,31]) In general, sleep quality and quantity decline with age and the prevalence of sleep disorder increases. [32] However, our data suggest that this pattern may not hold true in PD as younger participants reported *more* difficulty initiating and maintaining sleep, and *less* overall satisfaction with their sleep. Other authors have reported a similar pattern of results (e.g. cluster analysis revealed a different profile of sleep disorder associated with age in PD. [33,34] In light of these findings, one might expect more severe insomnia in younger patients, those with a shorter disease duration and patients who have recently commenced medication.

Scores from the sub-factor measuring OSA and RBD symptoms were higher in men than women. However, these scores did not correlate with any of the sample parameters tested. Both the OSA and RBD literatures report both greater prevalence and more florid symptomatic presentation in men. [35,36] Given that scores on this factor did not correlate with mood or neuroticism, response bias is less likely on this sub-factor, but the absence of expected correlations (for instance, increasing age and disease duration are both associated with higher prevalence of both disorders), [37] may also reflect low sensitivity and specificity of these items given that subtle symptoms of these disorders may be difficult to detect via self-report.

### Conclusions

Our bifactor analysis of the PDSS-R, whilst requiring independent replication, and further exploration in the PDSS and PDSS-2 has several practical implications: i) Variance from items measuring motor symptoms was wholly accounted for by the general factor. It seems likely that mood and personality influence the degree to which motor symptoms affect self-reported sleep quality (to a greater extent than disease-specific factors like disease duration and LED). It may be helpful to covary for affective symptoms and neuroticism in analysis; ii) Each item loading significantly onto a factor (either the general factor or a sub-factor), contributes unequal amounts of variance to this score (e.g. overt symptoms of OSA and RBD- witnessed apnoea and violence during sleep load more strongly on the specific sub-factor than less specific symptoms- snoring and distressing dreams). For this reason, there is a strong statistical justification for weighting item scores from the PDSS scales using factor loadings. These weighted scores are likely to yield more sensitive data; iii) Finally, we used the PDSS-R proposed by Tse et al. [2] Although its purpose and structure are similar to the PDSS and PDSS-2 and there is a high degree of content overlap between the three scales: the scales are not identical. Therefore, it will be important to test a similar, multidimensional model using PDSS and PDSS-2 data. Our choice of instrument was influenced by the fact that the PDSS-R includes four items quantifying OSA and RBD symptoms. These are, in our opinion, important inclusions in an omnibus scale of sleep disruption in PD and the PDSS-R may be a useful alternative to PDSS or the PDSS-2.

Given the extremely poor fit of the 1 factor CFA, in addition to systematic differences in item response across factors, we recommend caution in using summed PDSS total scores as a cardinal indicator of sleep in PD.

## References

[1] Chaudhuri KR, Pal S, Dimarco A, Bridgman K, Mathew R, Pezzela FR, et al The Parkinson’s disease sleep scale: a new instrument for assessing sleep and nocturnal disability in Parkinson’s disease. J Neurol Neurosurg Psychiatry. 2002;73: 629–635. doi: 10.1136/jnnp.73.6.629 12438461 10.1136/jnnp.73.6.629 PMC1757333

[2] Tse W, Liu Y, Barthlen GM, Halbig TD, Tolgyesi SV, Gracies J-M, et al Clinical usefulness of the Parkinson’s disease sleep scale. Parkinsonism Relat Disord. 2005;11: 317–321. doi: 10.1016/j.parkreldis.2005.02.006 15882956 10.1016/j.parkreldis.2005.02.006

[3] Trenkwalder C, Kohnen R, Högl B, Metta V, Sixel-Döring F, Frauscher B, et al Parkinson’s disease sleep scale-validation of the revised version PDSS-2. Mov Disord. 2011;26: 644–652. doi: 10.1002/mds.23476 21312275 10.1002/mds.23476

[4] Lee AH, Weintraub D. Psychosis in Parkinson’s disease without dementia: common and comorbid with other non-motor symptoms. Mov Disord. 2012;27: 858–63. doi: 10.1002/mds.25003 22674352 10.1002/mds.25003 PMC3511789

[5] Chaudhuri KR, Martinez-Martin P, Rolfe KA, Cooper J, Rockett CB, Giorgi L, et al Improvements in nocturnal symptoms with ropinirole prolonged release in patients with advanced Parkinson’s disease. Eur J Neurol. 2012;19: 105–113. doi: 10.1111/j.1468-1331.2011.03442.x 21699627 10.1111/j.1468-1331.2011.03442.x

[6] Honig H, Antonini A, Martinez-Martin P, Forgacs I, Faye GC, Fox T, et al Intrajejunal levodopa infusion in Parkinson’s disease: a pilot multicenter study of effects on nonmotor symptoms and quality of life. Mov Disord. 2009;24: 1468–1474. doi: 10.1002/mds.22596 19425079 10.1002/mds.22596

[7] Reise SP, Morizot J, Hays RD. The role of the bifactor model in resolving dimensionality issues in health outcomes measures. Qual Life Res. 2007;16: 19–31. doi: 10.1007/s11136-007-9183-7 17479357 10.1007/s11136-007-9183-7

[8] Chaudhuri KR, Pal S, DiMarco A, Whately-Smith C, Bridgman K, Mathew R, et al The Parkinson’s disease sleep scale: A new instrument for assessment of sleep, nocturnal disability and daytime sleepiness in Parkinson’s disease. J Neurol Neurosurg Psychiatry. 2002;73: 629–635. doi: 10.1136/jnnp.73.6.629 12438461 10.1136/jnnp.73.6.629 PMC1757333

[9] Kovács N, Horváth K, Aschermann Z, Ács P, Bosnyák E, Deli G, et al Independent validation of Parkinson’s disease Sleep Scale 2nd version (PDSS-2). Sleep Biol Rhythms. 2016;14: 63–73. doi: 10.1007/s41105-015-0024-8

[10] Miller IN, Cronin-Golomb A. Gender differences in Parkinson’s disease: Clinical characteristics and cognition. Mov Disord. 2010;25: 2695–2703. doi: 10.1002/mds.23388 20925068 10.1002/mds.23388 PMC3003756

[11] Morin AJS, Arens AK, Marsh HW, Arens K. A Bifactor Exploratory Structural Equation Modeling Framework for the Identification of Distinct Sources of Construct-Relevant Psychometric Multidimensionality. Struct Equ Model A Multidiscip J. 2015;5511: 1–24. doi: 10.1080/10705511.2014.961800

[12] Vorderwuelbecke, B. J. Breuer E, Kretz R, Grosse P. Is sleep related breathing disorder associated with REM sleep behavioural disorder in patients with idiopathic Parkinson’s disease? 22nd Congress European Sleep Research Society. Tallinn; 2014. p. 2649.

[13] Iranzo A, Santamaría J, Rye DB, Valldeoriola F, Martí MJ, Muñoz E, et al Characteristics of idiopathic REM sleep behavior disorder and that associated with MSA and PD. Neurology. 2005;65: 247–252. doi: 10.1212/01.wnl.0000168864.97813.e0 16043794 10.1212/01.wnl.0000168864.97813.e0

[14] Loftus AM, Bucks RS, Thomas M, Kane R, Timms C, Barker RA, et al Retrospective Assessment of Movement Disorder Society Criteria for Mild Cognitive Impairment in Parkinson’s Disease. J Int Neuropsychol Soc. 2015;21: 137–145. doi: 10.1017/S1355617715000041 25666735 10.1017/S1355617715000041

[15] Hughes AJ, Daniel SE, Kilford L, Lees AJ. Accuracy of clinical diagnosis of idiopathic Parkinson’s disease: a clinico-pathological study of 100 cases. J Neurol Neurosurg Psychiatry. 1992;55: 181–184. doi: 10.1136/jnnp.55.3.181 1564476 10.1136/jnnp.55.3.181 PMC1014720

[16] DeYoung CG, Quilty LC, Peterson JB. Between facets and domains: 10 aspects of the Big Five. J Pers Soc Psychol. 2007;93: 880–896. doi: 10.1037/0022-3514.93.5.880 17983306 10.1037/0022-3514.93.5.880

[17] Henry JD, Crawford JR. The short-form version of the Depression Anxiety Stress Scales (DASS-21): construct validity and normative data in a large non-clinical sample. Br J Clin Psychol. 2005;44: 227–239. 16004657 10.1348/014466505X29657

[18] Wang J, Wang X. Structural Equation Modelling: Applications Using Mplus. Wiley; 2012.

[19] Lee I, Preacher K. Calculation for the test of the difference between two dependant correlations with one variable in common [Internet]. 2013. http://quantpsy.org

[20] Trenkwalder C, Kies B, Rudzinska M, Fine J, Nikl J, Honczarenko K, et al Rotigotine effects on early morning motor function and sleep in Parkinson’s disease: A double-blind, randomized, placebo-controlled study (RECOVER). Mov Disord. 2011;26: 90–99. doi: 10.1002/mds.23441 21322021 10.1002/mds.23441 PMC3072524

[21] Norlinah MI, Afidah KN, Noradina aT, Shamsul aS, Hamidon BB, Sahathevan R, et al Sleep disturbances in Malaysian patients with Parkinson’s disease using polysomnography and PDSS. Parkinsonism Relat Disord. Elsevier Ltd; 2009;15: 670–4. doi: 10.1016/j.parkreldis.2009.02.012 19362875 10.1016/j.parkreldis.2009.02.012

[22] Stefansdottir S, Gjerstad MD, Tysnes OB, Larsen JP. Subjective sleep problems in patients with early Parkinson’s disease. Eur J Neurol. 2012;19: 1575–1581. doi: 10.1111/j.1468-1331.2012.03791.x 22747791 10.1111/j.1468-1331.2012.03791.x

[23] Neikrug AB, Maglione JE, Liu L, Natarajan L, Avanzino JA, Corey-Bloom J, et al Effects of Sleep Disorders on the Non-Motor Symptoms of Parkinson Disease. J Clin Sleep Med. 2013;9: 1119–29. doi: 10.5664/jcsm.3148 24235892 10.5664/jcsm.3148 PMC3805796

[24] Dhawan V, Dhoat S, Williams AJ, Dimarco A, Pal S, Forbes A, et al The range and nature of sleep dysfunction in untreated Parkinson’s disease (PD). A comparative controlled clinical study using the Parkinson’s disease sleep scale and selective polysomnography. J Neurol Sci. 2006;248: 158–62. doi: 10.1016/j.jns.2006.05.004 16780888 10.1016/j.jns.2006.05.004

[25] Fernandez-Mendoza J, Calhoun S, Bixler EO, Pejovic S, Karataraki M, Liao D, et al Insomnia with objective short sleep duration is associated with deficits in neuropsychological performance: a general population study. Sleep. 2010;33: 459–65. 20394314 10.1093/sleep/33.4.459 PMC2849784

[26] Gunn DG, Naismith SL, Lewis SJG. Sleep disturbances in Parkinson disease and their potential role in heterogeneity. J Geriatr Psychiatry Neurol. 2010;23: 131–137. doi: 10.1177/0891988709358591 20101072 10.1177/0891988709358591

[27] Bower JH, Brandon R. Grossardt DMM, Ahlskog JE, Colligan RC, Geda YE, Therneau TM, et al Anxious Personality Predicts an Increased Risk of Parkinson’s Disease. Mov Disord. 2010;25: 2105–2113. doi: 10.1002/mds.23230 20669309 10.1002/mds.23230 PMC3089895

[28] Dorsey CM, Bootzin RR. Subjective and psychophysiologic insomnia: An examination of sleep tendency and personality. Biol Psychiatry. 1997;41: 209–216. doi: 10.1016/0006-3223(95)00659-1 9018392 10.1016/0006-3223(95)00659-1

[29] Martinez-Martin P, Visser M, Rodriguez-Blazquez C, Marinus J, Chaudhuri KR, van Hilten JJ. SCOPA-sleep and PDSS: two scales for assessment of sleep disorder in Parkinson’s disease. Mov Disord. 2008;23: 1681–8. doi: 10.1002/mds.22110 18709672 10.1002/mds.22110

[30] Ancoli-Israel S, Ayalon L, Salzman C. Sleep in the elderly: normal variations and common sleep disorders. Harv Rev Psychiatry. 2008;16: 279–286. doi: 10.1080/10673220802432210 18803103 10.1080/10673220802432210

[31] Ohayon MM, Carskadon MA, Guilleminault C, Vitiello MV. Meta-analysis of quantitative sleep parameters from childhood to old age in healthy individuals: developing normative sleep values across the human lifespan. Sleep. 2004;27: 1255–73. Available: http://www.ncbi.nlm.nih.gov/pubmed/15586779 15586779 10.1093/sleep/27.7.1255

[32] Martin J, Shochat T, Ancoli-Israel S. Assessment and treatment of sleep disturbances in older adults. Clin Psychol Rev. 2000;20: 783–805. doi: 10.1016/S0272-7358(99)00063-X 10983268 10.1016/s0272-7358(99)00063-x

[33] van Rooden SM, Heiser WJ, Kok JN, Verbaan D, Van Hilten JJ, Marinus J. The identification of Parkinson’s disease subtypes using cluster analysis: A systematic review. Mov Disord. 2010;25: 969–978. doi: 10.1002/mds.23116 20535823 10.1002/mds.23116

[34] Verbaan D, van Rooden SM, Visser M, Marinus J, Van Hilten JJ. Nighttime sleep problems and daytime sleepiness in Parkinson’s disease. Mov Disord. 2008;23: 35–41. doi: 10.1002/mds.21727 17960797 10.1002/mds.21727

[35] Lin CM, Davidson TM, Ancoli-Israel S. Gender differences in obstructive sleep apnea and treatment implications. Sleep Med Rev. Elsevier Ltd; 2008;12: 481–496. doi: 10.1016/j.smrv.2007.11.003 18951050 10.1016/j.smrv.2007.11.003 PMC2642982

[36] Olson EJ, Boeve BF, Silber MH. Rapid eye movement sleep behaviour disorder: demographic, clinical and laboratory findings in 93 cases. Brain. 2000;123 (Pt 2): 331–9. Available: http://www.ncbi.nlm.nih.gov/pubmed/10648440 10648440 10.1093/brain/123.2.331

[37] Sixel-Döring F, Trautmann E, Mollenhauer B, Trenkwalder C. Age, drugs, or disease: What alters the macrostructure of sleep in Parkinson’s disease? Sleep Med. 2012;13: 1178–1183. doi: 10.1016/j.sleep.2012.06.009 22841842 10.1016/j.sleep.2012.06.009

